# Root nutrient capture and leaf resorption efficiency modulated by different influential factors jointly alleviated P limitation in *Quercus acutissima* across the North–South Transect of Eastern China

**DOI:** 10.48130/FR-2022-0007

**Published:** 2022-05-24

**Authors:** Ran Tong, Yuxiang Wen, Jingyuan Wang, Chenyang Lou, Cong Ma, Nianfu Zhu, Wenwen Yuan, G. Geoff Wang, Tonggui Wu

**Affiliations:** 1 East China Coastal Forest Ecosystem Long-term Research Station, Research Institute of Subtropical Forestry, Chinese Academy of Forestry, Hangzhou, Zhejiang 311400, China; 2 Department of Forestry and Environment Conservation, Clemson University, Clemson, SC29634-0317, United States of America

**Keywords:** NSTEC, Latitude, N:P ratio, *NuRE*, *RSAF*

## Abstract

Soil and climatic conditions are known to have close associations with plant morphological and stoichiometric traits at a regional scale along latitudinal gradients; however, how latitude drives biotic and abiotic factors affecting plant nutrient acquisition to accommodate environmental nutrient deficiency remains unclear. We quantified soil, root, leaf, and leaf litter nitrogen (N) and phosphorus (P) concentrations to determine the potentially limiting nutrient and the simultaneous responses of root capture and leaf resorption to nutrient deficiency in seven *Quercus acutissima* forests across the North–South Transect of Eastern China. The results showed that the mean leaf and root N:P ratios in *Q. acutissima* were 21.58 and 20.23, respectively, which markedly exceeded the P limitation threshold of 16 for terrestrial plants. The mean leaf litter N and P were 10.63 mg/g and 0.51 mg/g, respectively, indicating that P resorption proficiency was relatively higher than N resorption proficiency. N displayed higher stoichiometric homeostasis than P in the leaf. The leaf and root N:P ratios showed a quadratic variation that first decreased and then increased as latitude increased, whereas the phosphorus resorption efficiency and root-soil accumulation factor of P displayed the opposite trend. Partial least square path modeling ( *PLS-PM*) analysis demonstrated that root nutrient capture and leaf nutrient resorption were regulated by different influential factors. Overall, these findings provide new insights into plant strategies to adapt to environmental nutrient deficiency, as well as the scientific basis for predicting the spatial and temporal patterns of nutrient acquisition in the context of climate change.

## INTRODUCTION

Nitrogen (N) and phosphorus (P) are essential nutrient elements for plant growth, and their availability widely limits production formation in terrestrial ecosystems, restricting the improvement of the future terrestrial carbon sink ^[ 1, 2] ^. A traditional view is that plant growth is often limited by N at mid and high latitudes, and by P at low latitudes ^[ 3− 5] ^. Recent studies have shown that plant growth is co-limited by N and P mainly based on the reaction of partial plant biomass to exogenous N or P addition ^[ 6− 8] ^. Overall, given that nutrient deficiency is a widely existing environmental stress that can seriously affect plant growth and primary productivity, the identification of the plant growth nutrient limitation at a local or regional scale would help to correctly assess the terrestrial carbon sink under global change ^[ 1, 9] ^.

Plants have developed effective ways to deal with N or P limitation for maintaining normal life activities, and most of the nutrients supplied for plant growth are originally obtained through root uptake. Generally, in nutrient-deficient environments, the energetic cost typically increases in proportion to nutrient capture since a large quantity of roots are needed for successful patch exploitation ^[ 10] ^. Root capture strategy would be favored because the nutrients derived from root uptake become less expensive as soil nutrient availability increases ^[ 11] ^. In addition, plants have evolved some nutrient conservation mechanisms, i.e., leaf nutrient resorption, whereby some of the nutrients are transferred and preserved during leaf senescence to retain more critical nutrients to realize the goal of the adaptation to a more nutrient-barren environment ^[ 12] ^. As has been widely reported, leaf resorption is highly dependent on the soil nutrient availability, i.e., leaf nitrogen resorption efficiency ( *NRE*) generally decreases with increased soil N availability ^[ 13− 15] ^.

The allocation between root capture and leaf resorption is strongly determined by the strength and type of nutrient limitation, with diverse response strategies to changes in nutrient availability in the surrounding environment ^[ 16, 17] ^. However, it remains unclear whether the response of root capture and leaf resorption to changes in light, water, heat, and soil conditions occurs at regional or larger scales. A continuous three-year observation demonstrated that the amount of active N deposition exceeded the critical loads of forest ecosystems along the North–South Transect of Eastern China (NSTEC) ^[ 18] ^, which might reduce the N limitation but exacerbate the P limitation of plant growth, especially in mature subtropical forests ^[ 2, 19, 20] ^.

In this study, we selected mature *Q. acutissima* forests as the research subject and set up seven sampling sites in the central part of the NSTEC, mainly in the northern and middle subtropical area of China, along with various levels of heat, rainfall and ultraviolet intensity. We aimed to determine the type of nutrient limitation for plant growth, as well the latitudinal patterns and environmental drivers of root capture and leaf resorption. More specifically, we tested two hypotheses: (1) plant growth was limited by P; (2) root nutrient capture and leaf nutrient resorption both alleviated the P limitation, regulated by different influential factors along a latitudinal gradient. In general, we expected to clarify the nutrient acquisition patterns of plant leaf and root tissues in response to potential soil nutrient deficiency and the underlying mechanisms, which should help expand existing knowledge on the ability of plants to adapt to environmental stress.

## RESULTS

### Description of potential limitation for plant growth

**Figure 1:**
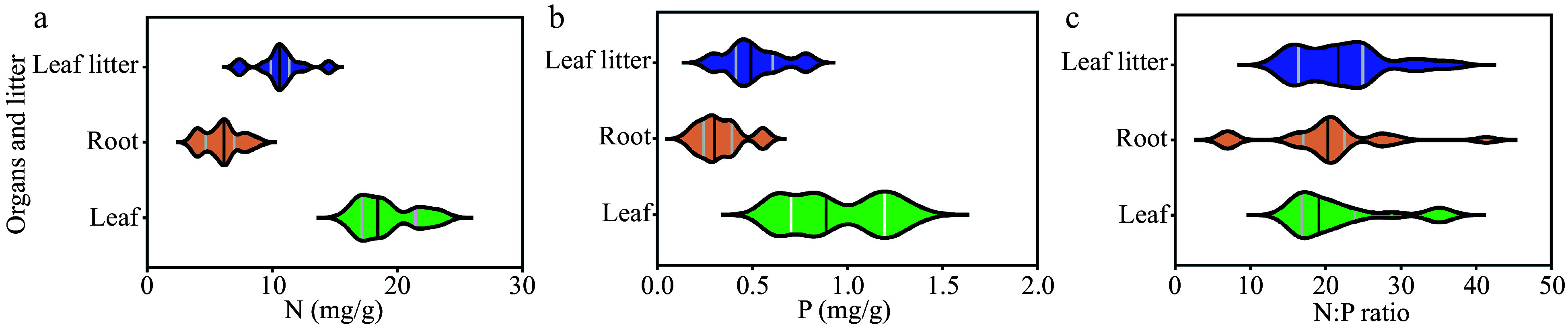
Description of the leaf, root and litter N and P stoichiometry *(n* = 21). N, nitrogen; P, phosphorus; N:P ratio, the ratio of nitrogen and phosphorus.

In each sampling plot, we collected leaves, roots and leaf litters from each sample tree and mixed them to obtain one leaf, root, and leaf litter sample. We obtained 21 samples in total for each type of tissues from the 21 sampling plots. We used violin plots to represent the median value and the variation ranges of 21 leaf, root, and leaf litter samples ( Fig. 1). The median values of leaf N and P concentrations were 18.40 mg/g and 0.89 mg/g, and the coefficients of variation (CV) were 12.23% and 27.37%, respectively. The median values of root N and P concentrations were 6.14 mg/g and 0.30 mg/g, and the CVs were 24.71% and 36.36%, respectively. The median values of leaf and root N:P ratios were 19.12 and 20.31, ranging from 14.84 to 36.01 and from 6.71 to 41.35, respectively. The median values of leaf litter N, P concentrations and N:P ratio were 10.60 mg/g, 0.49 mg/g and 21.69, respectively.

As all the data fit an approximate normal distribution, we also calculated the arithmetic means of the 21 samples of roots, leaves, and leaf litters to compare with the commonly-used thresholds that reflect the potential limitation for plant growth. The mean leaf N and P concentrations were 19.13 mg/g and 0.95 mg/g, respectively. The mean root N and P concentrations were 5.99 mg/g and 0.33 mg/g, respectively. The mean leaf and root N:P ratios were 21.58 and 20.23, respectively. The mean leaf litter N, P concentrations and N:P ratio were 10.63 mg/g, 0.51 mg/g and 22.16, respectively. In general, the leaf resorption proficiency and efficiency suggested that the P concentration could not meet the demand of plant growth.

**Figure 2:**
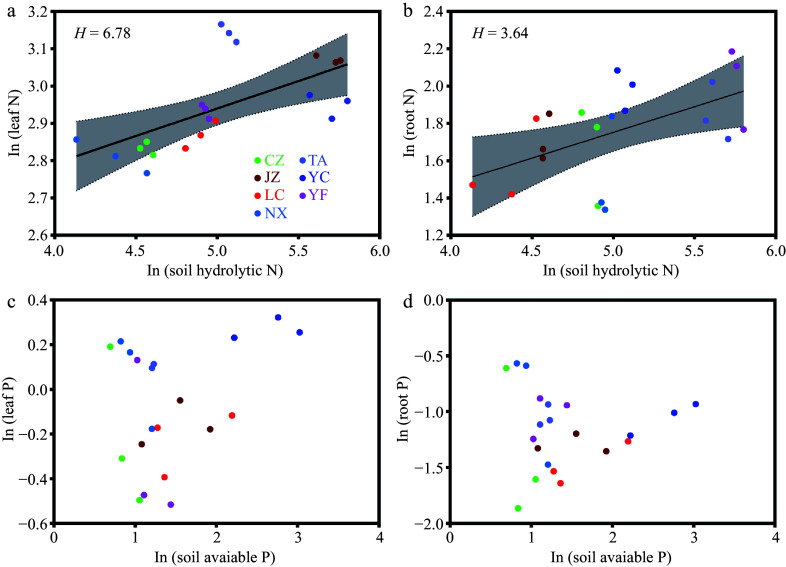
Relationships between N and P in the leaf and root, and soil available nutrient values ( *n* = 21). Different color dots represent samples from different sampling sites. More specifically, LC, Lechang, Guangdong Province; JZ, Jingzhou, Hunan Province; YF, Yifeng, Jiangxi Province; CZ, Chuzhou, Anhui Province; YC, Yichang, Hubei Province; NX, Neixiang, Henan Province; TA, Tai'an, Shandong Province.

We calculated the homoeostatic coefficients for nitrogen ( *H*_*N*_) and phosphorus ( *H*_*P*_) of leaves and roots using the measured data from the plots at seven *Q. acutissima* sites. The *H*_*N*_ values for the leaf and root samples were 6.78 and 3.64, respectively ( Fig. 2a, b). The *H*_*P*_ values for the leaf and root samples were not determined ( Fig. 2c, d). The above results show that *Q. acutissima* has stronger regulation ability of N than of P.

### Latitudinal patterns of nutrient limitation and nutrient acquisition

**Figure 3:**
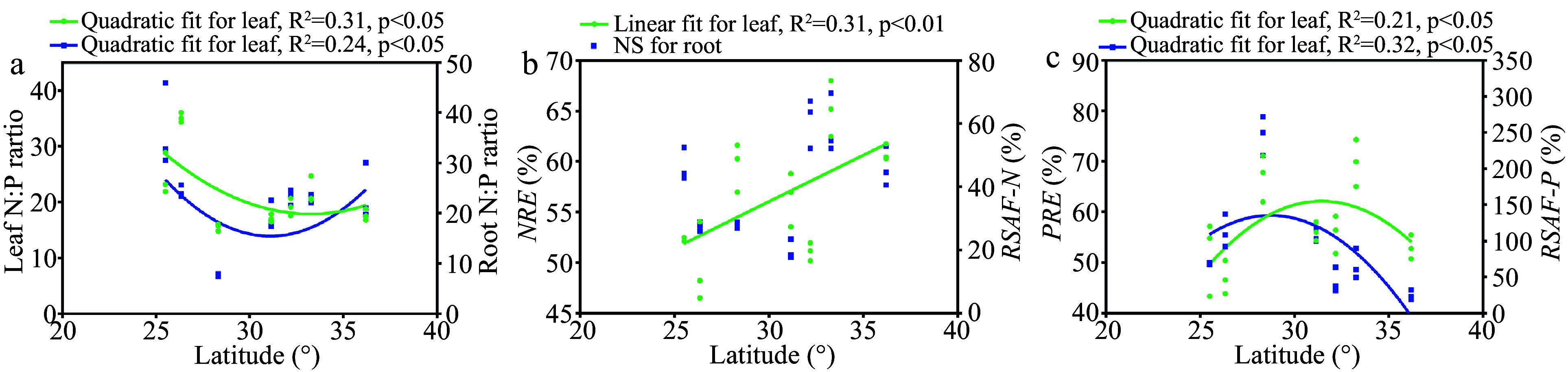
Trends of nutrient limitation and nutrient acquisition with latitude ( *n* = 21). *NRE*, nitrogen resorption efficiency; *PRE*, phosphorus resorption efficiency; *RSAF-N*, root-soil accumulation factor of nitrogen; *RSAF*-P, root-soil accumulation factor of phosphorus.

We investigated the latitudinal patterns of the leaf and root N:P ratios, nutrient resorption efficiency ( *NuRE*), and root-soil accumulation factor ( *RSAF*). Leaf and root N:P ratios decreased with latitude up to 31.0° N and the increased with latitude above 31.0° N ( Fig. 3a). *NRE* showed a tendency to increase with the latitude, while *RSAF* of N ( *RSAF-N)* showed no significant trend ( Fig. 3b). P resorption efficiency ( *PRE*) and *RSAF* of P ( *RSAF-P*) increased as the latitude increases when the latitude was below 31.0° N but decreased when the latitude was higher than 31.0° N ( Fig. 3c). Overall, we observed roughly opposite latitudinal trends between P limitation and P acquisition.

### Associations between latitude and nutrient acquisition based on partial least square path modeling ( *PLS-PM*)

**Figure 4:**
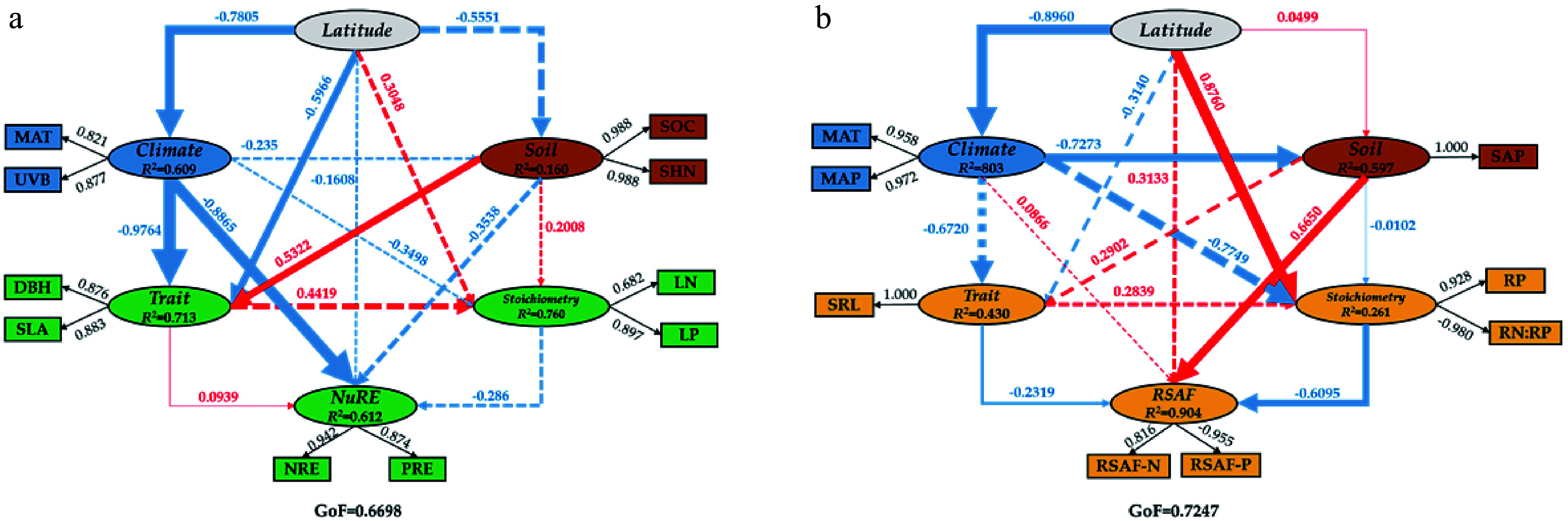
Predicted partial least squares path modeling ( *PLS-PM*) for the direct and indirect associations between latitude and nutrient acquisition *(n* = 21). Ellipses represent the structural model, and boxes represent corresponding measurement models. In the structural model, the lines indicate paths, and the values adjacent to the lines denote the magnitude of the path coefficients calculated by PLS regression. R ^2^ values are shown for all endogenous latent variables in the ellipses. Values in the measurement model represent the loadings between a latent variable and its indicators. The figure shows the final models after model diagnostic processes. All values in the measurement model exceeded 0.70. The pseudo goodness-of-fit (GoF) of the model was 0.6698 for the association between latitude and *NuRE* and 0.7274 for the association between latitude and *RSAF*, implying that the predicted models fit well. LAT, latitude; LON, longitude; ALT, altitude; MAP, mean annual precipitation; MAT, mean annual temperature; UVB, type B ultraviolet radiation; SOC, soil organic carbon; SHN, soil hydrolytic nitrogen; SAP, soil available phosphorus; SN:P, soil hydrolytic nitrogen to soil available phosphorus ratio. DBH, diameter at breast height; H, height; SLA, specific leaf area.

*PLS-PM* was used to evaluate the associations among latitude, climatic variables, soil nutrient conditions, tree morphological traits, organ N and P stoichiometry, and nutrient acquisition by pooling together all collected data from 21 sampling sites. The *PLS-PM* analysis showed that latitude had no direct association (path coefficient = −0.518, *p* < 0.01) with *NuRE* (including *NRE* and *PRE*) and *RSAF* ( *RSAF*- *N* and *RSAF*- *P*) ( Fig. 4a, b). For the indirect association between latitude and *NuRE*, latitude had a significantly negative association (path coefficient = −0.7805, *p* < 0.001) with climate (finally including MAT and UVB), and *Climate* had a negative association (path coefficient = −0.8865, *p* < 0.001) with *NuRE* ( Fig. 4a). The significant association between latitude and *NuRE* for significant paths was 0.692 (−0.7805 × 0.8865). In total, 61.2% of the *NuRE* variance was explained with five other influential factors in the model ( Fig. 4a).

For the indirect associations between latitude and *RSAF*, latitude had a significantly negative association (path coefficient = −0.8960, *p* < 0.001) with *Climate* (finally including MAT and MAP), *Climate* had a negative association (path coefficient = −0.7273, *p* < 0.001) with *Soil* (finally including soil available phosphorus, SAP), and *Soil* had a positive association (path coefficient = −0.6650, *p* < 0.001) with *RASF* ( Fig. 4b). Moreover, latitude had a significant positive association (path coefficient = 0.8760, *p* < 0.001) with *Stoichiometry* (finally including RP and RN:RP), and *Stoichiometry* had a negative association (path coefficient = −0.6095, *p* < 0.001) with *RASF*. The significant association between latitude and *RSAF* for significant paths was 0.101 (−0.8960 × −0.7273 × 0.6650 + 0.8760 × −0.6095). In total, 90.38% of the *RSAF* variance was explained with five other influential factors in the model ( Fig. 4b).

## DISCUSSION

### Plant growth of *Q. acutissima* exhibited P limitation

In view of the importance of nutrient limitation for tree growth and productivity, as well as the carbon sink enhancement in forest ecosystems, methods to determine the limitation of plant growth from an ecological perspective have been widely developed. Among them, plant tissue N:P ratios are widely applied to indicate soil nutrient availability and limitation. In this study, the leaf N:P ratio was 21.58, which was markedly higher than the frequently used P-limitation threshold (16) for terrestrial ecosystems ^[ 21] ^. The root N:P ratio was 20.23, which was much higher than that for China (14.27) and globally (16.0) ^[ 22, 23] ^. According to the widely accepted view that a higher N:P ratio indicated more critical P limitation, the root N:P ratio might also play an indicator role in the P limitation of *Q. acutissima.* These results provided initial support for our first hypothesis.

Similarly, the efficiency or proficiency of nutrient resorption in leaves during senescence is considered an indicator of plant adaptation to nutrient deficiency ^[ 24, 25] ^. Based on nutrient concentrations in senesced leaves, Killingbeck ^[ 26]^ argued that > 10 mg/g N represented incomplete resorption of N across 87 species, and < 0.5 mg/g P represented complete resorption of P in deciduous species. In this study, the mean leaf litter N and P concentrations were 10.63 mg/g and 0.51 mg/g, respectively, indicating that the potential resorption of P was higher than that of N during *Q. acutissima* senescence. This suggested that the growth of *Q. acutissima* was more limited by P than by N *,* also supporting the first hypothesis. Moreover, the differences in *NRE* and *PRE* can be used to describe the type of nutrient limitation, i.e., *PRE*> *NRE* indicates a P limitation. However, in our study, the *NRE* (56.43%) and *PRE* (57.20%) were similar ( *p* > 0.05; Supplemental Fig. S1a), which largely does not support the first hypothesis. On the contrary, *RSAF-P* was markedly higher than *RSAF-N* ( *p* < 0.001; Supplemental Fig. S1b), suggesting that the P demand of *Q. acutissima* was higher than that of N.

The ability of plants to maintain a stable nutrient composition regardless of changes in environmental nutrients could also be used as an indicator for the evaluation of plant growth restriction, which is usually described as stoichiometric homeostasis ^[ 27, 28] ^. Our results showed that *H*_*N*_ values were determined for the leaf and root, while *H*_*P*_ values were not, suggesting that *Q. acutissima* might have stronger N than P regulation ability. This result is in accordance with the stability of limiting elements hypothesis, which also indirectly supports our first hypothesis.

### Latitudinal patterns and environmental drivers of nutrient acquisition and nutrient limitation in *Q. acutissima*

The exploration of biogeographical patterns of nutrient traits would deepen our understanding of how plants respond to environmental changes along a latitudinal gradient dominated by heat or a longitudinal gradient dominated by rainfall. For instance, the observation in the NSTEC showed that vegetation water and nutrient use efficiencies in natural forest ecosystems were dominantly driven by climate and were significantly affected by soil nutrient factors along the latitudinal gradient ^[ 29] ^. Generally, nutrient traits exhibit a linear pattern along a specific gradient of environmental change. However, non-linear patterns are also observed due to the difference in study scope, as well as the species specificity of biogeochemical niches ^[ 30] ^. In this study, the leaf and root N:P ratios both exhibited initial increasing and then subsequent decreasing trends, indicating that P limitation was less severe in the central distribution area. Consistent with the description of the second hypothesis, our results showed that *PRE* and *RSAF-P* presented an increasing and then a decreasing trend before and after 31.0° N, playing common relief roles in mediating the response to P limitation. Thus, our field investigation at a regional scale provided valuable evidence that different types of nutrient acquisition in plants might jointly contribute to the release of nutrient limitation for plant growth.

The large changes in climatic and edaphic factors brought about by regional variation of latitude have been widely confirmed to play regulatory roles in the variation of plant morphological and stoichiometric traits, which greatly improved our knowledge of plant adaptation to environmental changes ^[ 31, 32] ^. However, the response modes of nutrient acquisition to environmental changes along the latitudinal gradient have not been extensively explored. In the current study, direct and indirect associations between *Latitude* and *NuRE* or *RSAF* were observed from the *PLS-PM* analysis. Similarly, the results showed that latitude had no significantly direct effect on *NuRE* and *RSAF* but did have significantly indirect effects through different pathways.

In the two *PLS-PM* models, *Latitude* had significant direct effects on *Climate*, which has been widely observed in other studies conducted at national or global scales ^[ 33] ^. In the model for the associations between *Latitude* and *NuRE*, *Climate* showed significantly negative effects on *NuRE*, whereas *Climate* first affected *Soil* and then *RSAF* in the model for the associations between *Latitude* and *RSAF*, which confirmed the second hypothesis. For the appearance of the above different pathways of the associations between *Latitude* and *NuRE* and *RSAF*, we speculated that UVB was the key influential factor because it is closely related to leaf N and P stoichiometric traits ^[ 34] ^. Furthermore, the results showed that *Latitude* had significant effects on root *stoichiometry*, which is not in line with Liu et al. ^[ 35] ^, who found that fine root N and P concentrations did not increase with increasing latitude for *Q. variabilis* along a south–north transect in China. We also found a negative association between root stoichiometry and *RSAF*, also indicating that a high root N:P ratio corresponded to the low *RSAF-P*, partly supporting our second hypothesis.

## CONCLUSIONS

In this study, we carried out a field investigation and explored the response of nutrient acquisition to environmental nutrient deficiency along a latitudinal gradient. The results showed that the growth of *Q. acutissima* was generally limited by P, and the two different nutrient-acquisition strategies played joint roles in relieving P limitation while being regulated by diverse response pathways. In general, our findings confirmed the two proposed hypotheses, providing a scientific basis for exploring the self-regulation ability of *Q. acutissima* along latitudinal gradients, as well as the plant adaptation strategy to environmental nutrient deficiency under global change.

## MATERIALS AND METHODS

### Study area

The NSTEC is the 15th standard transect established by the International Geosphere–Biosphere Program (IGBP), which is a clearly heat-driven standard transect and a 'natural laboratory' to explore vegetation patterning, plant traits and ecosystem functioning, as well as their responses to environmental changes ^[ 36, 37] ^. Seven sampling sites were selected according to the distribution of *Q. acutissima* forests in the central part of NSTEC (Supplemental Fig. S2). The geographic position (including latitude, longitude and altitude) of each site was recorded. The soil available P contents in most of the sampling sites were less than 10 mg/kg (except Tai'an), which is usually regarded as the threshold of P deficiency for restricting plant growth (Supplemental Table S1). Thirty-year (1970–2000) mean annual temperature (MAT) and mean annual precipitation (MAP) for each site were derived from the WorldClim 1.4 database ( http://www.worldclim.org), a global dataset with a spatial resolution of c. 1 km ^2^. We also extracted the annual UVB radiation data from glUV ( http://www.ufz.de/gluv), a global data set of surface UVB radiation that represents long-term averages of UVB conditions with a 15-arc minute spatial resolution ^[ 34] ^.

### Sampling of leaves, roots, leaf litters and soil

The *Q. acutissima* sampling stands were selected based on the following criteria: at least 20-year-old mature forests with a diameter at breast height (DBH) > 15 cm and height (H) > 10 m as Liu et al. ^[ 38]^ described; no significant disturbance in the last 30 years; and good tree growth without the occurrence of pests and diseases. *Q. acutissima* was the dominant species in the tree layer, and the shrub and herbaceous layers were sparse at all sampling sites.

In September 2017, three plots (20 m × 20 m) were established at a similar altitude with the same slope direction, and all were marked for organ and leaf litter sampling at each sampling site; the distance between neighboring plots exceeded 20 m. The DBH and H were measured for all trees in each plot. Based on DBH and H, three average growth trees in each plot were selected. For each sample tree, fully expanded leaves from the upper and outer part of tree crowns were sampled, and the absorptive fine roots (the most distal two-order roots, < 2 mm) were dug out by excavation from five locations below the canopy in the 0−20 cm soil layer ^[ 39] ^. Three litter traps were set in each plot at the end of October 2017. Between November and December 2017, leaf litter was collected from three litter traps (1 m × 1 m size) in each plot.

Within each plot, soil samples were collected at five randomly selected locations. At each location, after removal of the litter layer, a soil core (0−20 cm) was collected with a 2.5-cm-diameter soil auger. The soil samples from each plot were combined into a composite soil sample, placed in a soil sample bag and transported to the laboratory at room temperature.

### Sample preparation and chemical analyses

The leaf area and root length were measured and analyzed by a scanner and ImageJ software (The National Institutes of Health NIH, Bethesda, MD, USA). The dry weight of the plant samples was determined by weighing after drying at 75°C for 48 h. The specific leaf area (SLA) was calculated as the ratio of the leaf area to the leaf dry weight, and the specific root length (SRL) was calculated as the ratio of the root length to the dry weight.

Fine roots (< 2 mm) were cleaned carefully to remove soil particles, using deionized water, and were dried in air. Leaf and root samples were oven-dried at 75 °C to a constant weight and were ground for further analysis. Soil samples were air dried in a shaded and ventilated environment. All samples were sieved through a 60-mesh sieve (0.25 mm in diameter) for chemical analysis.

Leaf, root and leaf litter N and P concentrations were determining for each sample using micro-Kjeldahl digestion followed by colorimetric determination on a flow injection auto-analyzer. The soil N concentration was determined by the Kjeldahl method, a continuous flow analyzer and element analyzer (Nitrogen determination methods of forest soils, LY/T 1228-2015). The soil P concentration was determined by the alkali melting method and acid dissolving method (Phosphorus determination methods of forest soils, LY/T 1323-2015).

### Data analyses

The nutrient resorption efficiency ( *NuRE*) was estimated as the percent difference between nutrient concentrations in the leaf litter and leaf, expressed as equation 1:

$ NuRE=\left(1-\frac{{\left[\mathrm{N}\mathrm{u}\right]}_{litter}}{{\left[\mathrm{N}\mathrm{u}\right]}_{leaf}}\times MCLF\right)\times 100{\%} $

where $ {\left[\mathrm{N}\mathrm{u}\right]}_{leaf} $ and $ {\left[\mathrm{N}\mathrm{u}\right]}_{litter} $ are the nutrient concentrations in the leaf and leaf litter, respectively, and *MCLF* is a mass loss correction factor with a value of 0.784 for deciduous angiosperm tree species ^[ 40] ^.

The root-soil accumulation factors ( *RSAF*) of N ( *RSAF-N*) and P ( *RSAF-P*) were defined as the ratio of nutrient concentrations in absorptive fine roots and soil ^[ 16] ^, expressed as equation 2:

$ RSAF=\frac{{\left[\mathrm{N}\mathrm{u}\right]}_{AFR}}{{\left[\mathrm{N}\mathrm{u}\right]}_{S oil}} $

where $ {\left[\mathrm{N}\mathrm{u}\right]}_{AFR} $ is the nutrient concentration of absorptive fine root (the most distal two-order roots); $ {\left[\mathrm{N}\mathrm{u}\right]}_{S oil} $ is the concentration of available nutrients at 0–20 cm.

The normality of all numerical variables, including N and P stoichiometry of organs, leaf litter, soil, *NuRE*, *RSAF*, MAT, MAP, UVB, SLA, SRL, DBH, and H, was checked by the Kolmogorov–Smirnov test, and Levene's test was used to examine the equality of the variances. When necessary, log transformations were used in order to meet the assumptions of normality and linearity. Linear regression analysis and polynomial regression analysis were performed to test the relationships between latitude and N, P stoichiometry, as well as root nutrient capture and leaf nutrient resorption. *PLS-PM* analysis was used to examine the effects of environmental and biological factors on root nutrient capture and leaf nutrient resorption. All analyses were performed using the R statistical platform 4.0.5 (R Development Core Team, www.R-project.org).

## SUPPLEMENTARY DATA

Supplementary data to this article can be found online.

## Supplementary Information

accompanies this paper at ( http://www.maxapress.com/article/doi/10.48130/FR-2022-0007)
