# Supplementary material for: Root nutrient capture and leaf resorption efficiency modulated by different influential factors jointly alleviated P limitation in Quercus acutissima across the North–South Transect of Eastern China
Source: For Res (Fayettev). 2022 May 24;2:7. doi: 10.48130/FR-2022-0007 (PMC11524281; doi:10.48130/FR-2022-0007)

Figure S2 Distribution of sampling sites of *Quercus acutissima* in NSTEC. Abbreviations of the site names were given as Table S1 showed.

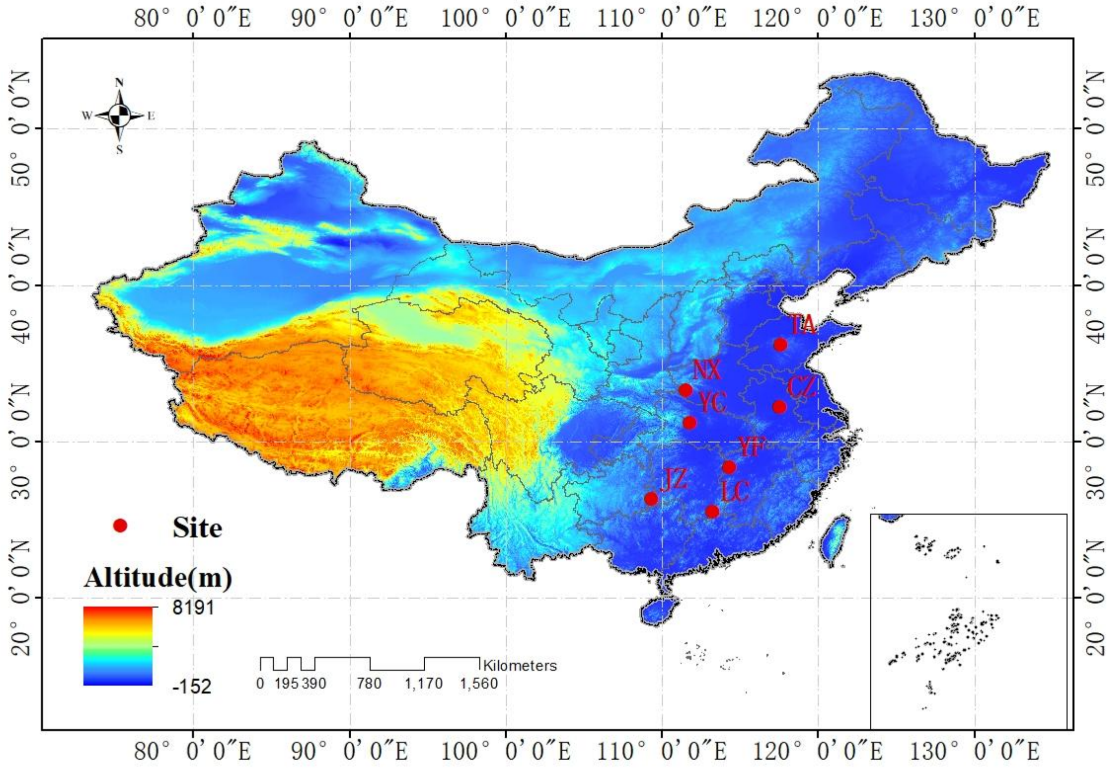

Supplement: Supplementary file 1 — Supplementary data to this article can be found online. [file FR-2022-0007-S1.zip › 10.48130_FR-2022-0007-Suppl-FigureS2.pdf]
