# Supplementary material for: Root nutrient capture and leaf resorption efficiency modulated by different influential factors jointly alleviated P limitation in Quercus acutissima across the North–South Transect of Eastern China
Source: For Res (Fayettev). 2022 May 24;2:7. doi: 10.48130/FR-2022-0007 (PMC11524281; doi:10.48130/FR-2022-0007)
Supplement: Supplementary file 1 — Supplementary data to this article can be found online. [file FR-2022-0007-S1.zip › 10.48130_FR-2022-0007-Suppl-TableS1.pdf]

Table S1 Location, climate and soil nutrient conditions of sampling sites

Sites	LAT	LON	ALT	MAP	MAT	UVB	SHN	SAP	SN:P
	(°)	(°)	(m)	(mm)	(℃)	(μW/cm^2)	(mg kg^{-1})	(mg kg^{-1})	
LC	25.50	113.29	400	1477.70	19.60	4831.99	134.33±12.50	2.84±0.52	47.84±4.51
JZ	26.34	109.36	495	1250.00	16.70	4601.75	299.33±23.25	3.43±0.69	88.96±13.30
YF	28.33	114.34	480	1767.00	17.20	4382.99	138.00±3.00	2.28±0.28	61.20±6.73
CZ	32.19	117.58	130	1074.65	15.90	3922.07	96.20±3.80	6.85±2.12	15.17±5.50
YC	31.16	111.80	1200	1161.05	16.70	3995.48	298.00±34.60	3.19±0.35	94.95±21.09
NX	33.28	111.53	620	761.05	15.00	3667.86	79.35±16.95	3.48±0.48	23.47±8.07
TA	36.20	117.60	740	685.60	12.80	3245.39	159.50±7.50	15.20±5.72	11.57±4.39

LC, Lechang, Guangdong Province; JZ, Jingzhou, Hunan Province; YF, Yifeng, Jiangxi Province; CZ, Chuzhou, Anhui Province; YC, Yichang, Hubei Province; NX, Neixiang, Henan Province; TA, Tai'an, Shandong Province; LAT, latitude; LON, longitude; ALT, altitude; MAP, mean annual precipitation; MAT, mean annual temperature; UVB, ultraviolet radiation b; SHN, soil hydrolytic nitrogen; SAP, soil available phosphorus; SN:P, soil hydrolytic nitrogen to soil available phosphorus ratio.
